# Dynamic PET evaluation of elevated FLT level after sorafenib treatment in mice bearing human renal cell carcinoma xenograft

**DOI:** 10.1186/s13550-016-0246-z

**Published:** 2016-12-12

**Authors:** Naoyuki Ukon, Songji Zhao, Wenwen Yu, Yoichi Shimizu, Ken-ichi Nishijima, Naoki Kubo, Yoshimasa Kitagawa, Nagara Tamaki, Kei Higashikawa, Hironobu Yasui, Yuji Kuge

**Affiliations:** 1 Department of Tracer Kinetics & Bioanalysis, Graduate School of Medicine, Hokkaido University, Kita 15 Nishi 7, Kita-ku Sapporo, 060-8638 Japan; 2 Central Institute of Isotope Science, Hokkaido University, Kita 15 Nishi 7, Kita-ku Sapporo, 060-0815 Japan; 3 Department of Molecular Imaging, Graduate School of Medicine, Hokkaido University, Kita 15 Nishi 7, Kita-ku Sapporo, 060-8638 Japan; 4 Department of Oral Diagnosis and Medicine, Graduate School of Dental Medicine, Hokkaido University, Kita 13 Nishi 7, Kita-ku Sapporo, 060-8638 Japan; 5 Department of Integrated Molecular Imaging, Graduate School of Medicine, Hokkaido University, Kita 15 Nishi 7, Kita-ku Sapporo, 060-8638 Japan; 6 Faculty of Pharmaceutical Sciences, Hokkaido University, Kita 12 Nishi 6, Kita-ku Sapporo, 060-0812 Japan; 7 Department of Nuclear Medicine, Graduate School of Medicine, Hokkaido University, Kita 15 Nishi 7, Kita-ku Sapporo, 060-8638 Japan

**Keywords:** Sorafenib, Tumor proliferation, 3′-[^18^F]fluoro-3′-deoxythymidine ([^18^F]FLT), Dynamic PET, Renal cell carcinoma xenograft

## Abstract

**Background:**

Sorafenib, an oral multikinase inhibitor, has anti-proliferative and anti-angiogenic activities and is therapeutically effective against renal cell carcinoma (RCC). Recently, we have evaluated the tumor responses to sorafenib treatment in a RCC xenograft using [Methyl-^3^H(N)]-3′-fluoro-3′-deoxythythymidine ([^3^H]FLT). Contrary to our expectation, the FLT level in the tumor significantly increased after the treatment. In this study, to clarify the reason for the elevated FLT level, dynamic 3′-[^18^F]fluoro-3′-deoxythymidine ([^18^F]FLT) positron emission tomography (PET) and kinetic studies were performed in mice bearing a RCC xenograft (A498).

The A498 xenograft was established in nude mice, and the mice were assigned to the control (*n* = 5) and treatment (*n* = 5) groups. The mice in the treatment group were orally given sorafenib (20 mg/kg/day p.o.) once daily for 3 days. Twenty-four hours after the treatment, dynamic [^18^F]FLT PET was performed by small-animal PET. Three-dimensional regions of interest (ROIs) were manually defined for the tumors. A three-compartment model fitting was carried out to estimate four rate constants using the time activity curve (TAC) in the tumor and the blood clearance rate of [^18^F]FLT.

**Results:**

The dynamic pattern of [^18^F]FLT levels in the tumor significantly changed after the treatment. The rate constant of [^18^F]FLT phosphorylation (k_3_) was significantly higher in the treatment group (0.111 ± 0.027 [1/min]) than in the control group (0.082 ± 0.009 [1/min]). No significant changes were observed in the distribution volume, the ratio of [^18^F]FLT forward transport (K_1_) to reverse transport (k_2_), between the two groups (0.556 ± 0.073 and 0.641 ± 0.052 [mL/g] in the control group).

**Conclusions:**

Our dynamic PET studies indicated that the increase in FLT level may be caused by the phosphorylation of FLT in the tumor after the sorafenib treatment in the mice bearing a RCC xenograft. Dynamic PET studies with kinetic modeling could provide improved understanding of the biochemical processes involved in tumor responses to therapy.

## Background

Tumor proliferation is a hallmark of the cancer phenotype and is one of the useful markers for evaluating the therapeutic effect and prognosis after conventional therapy in clinical oncology. Positron emission tomography (PET) using 3′-[^18^F]fluoro-3′-deoxythymidine ([^18^F]FLT) is one of the noninvasive methods of assessing tumor proliferation [1–3]. Accordingly, we have recently evaluated the tumor responses to sorafenib treatment in our model experiments using a renal cell carcinoma (RCC) xenograft and [methyl-^3^H(N)]-3′-fluoro-3′-deoxythythymidine ([^3^H]FLT) [4]. Sorafenib is a multikinase inhibitor that has anti-proliferative and anti-angiogenic activities and is reported to show significant therapeutic effects against RCC [5, 6]. Contrary to our expectation, however, the level of [^3^H]FLT in the tumor significantly increased after the sorafenib treatment [4].

Including in our previous study [4], static parameters such as standardized uptake value (SUV) have been extensively used to monitor treatment-induced changes in oncology [7–10]. PET is, however, a dynamic imaging modality that allows the imaging and observation of the distribution of a radiolabeled tracer throughout the body over time [11–15]. Therefore, tracer kinetic modeling techniques with PET have been widely used in neurology and cardiology to study biochemical and physiological processes in humans and small animals. Application of the kinetic modeling techniques could also provide improved understanding of the complex biochemical processes involved in tumor responses to therapy. Accordingly, in this study, to clarify the reason for the elevated FLT level after sorafenib treatment, we applied the kinetic modeling techniques and evaluated the dynamic patterns of [^18^F]FLT level in the tumors of the mice bearing a RCC xenograft (A498).

## Methods

### Radiopharmaceuticals

[^18^F]FLT was obtained from the Hokkaido University Hospital Cyclotron Facility, which was synthesized by standard procedures [16].

### Animal models

The entire experimental protocols were approved by the Laboratory Animal Care and Use Committee of Hokkaido University (approval number 13-0057) and performed in accordance with the Guidelines for Animal Experiments at the Graduate School of Medicine, Hokkaido University. Eight-week-old male BALB/c athymic nude mice were supplied by Japan SLC, Inc. (Hamamatsu, Japan) and used in all experiments. The room temperature was maintained between 23 and 25 °C, and the relative humidity was maintained between 45 and 60%. The institutional laboratory housing provided a 12-h light/dark cycle and met all the criteria of the Association for Assessment and Accreditation of Laboratory Animal Care (AAALAC) International. A RCC xenograft model was established using a human clear cell RCC (A498) cell line (European Collection of Cell Cultures, Salisbury, UK). A489 cells were maintained in RPMI-1640 medium (Invitrogen/Thermo Fisher Scientific, Inc., Carlsbad, CA, USA); supplemented with 10% fetal bovine serum, penicillin–streptomycin, and 0.03% glutamine; and incubated in an atmosphere of 5% CO_2_ and 95% air at 37 °C. A498 cells (1 × 10^7^ cells/0.1 mL) were subcutaneously inoculated into the right dorsal area of each mouse. When the tumors grew 12–13 mm in diameter, the mice were assigned to the control (*n* = 5) and sorafenib-treated (*n* = 5) groups. The mice in the sorafenib-treated group were administered sorafenib (20 mg/kg/day p.o.; Nexavar, Bayer Pharmaceuticals Corporation, West Haven, CT, USA) once daily for 3 days. The vehicle was administered to the control group [4].

### PET study

Twenty-four hours after the last treatment to reproduce the experimental conditions with our previous study [4, 17] and to prevent the acute influence after the treatment, we performed dynamic [^18^F]FLT PET using a small-animal multimodality system (Inveon, Siemens Medical Solutions, Knoxville, TN). The PET component consists of 1.5 × 1.5 × 10 mm^3^ lutetium oxyorthosilicate crystal elements with a ring diameter of 16.1 cm, to give an effective transaxial field of view (FOV) of 10 cm and an axial FOV of 12.7 cm. CT was also performed for attenuation correction. The mice were maintained under anesthesia with 1.0–1.5% isoflurane and placed on a heating sheet during scanning. Dynamic (list-mode) [^18^F]FLT PET was performed up to 120 min after the injection with [^18^F]FLT (7.63 ± 1.87 MBq bolus via the tail vein). The images were reconstructed and corrected for attenuation and scattered using the Fourier rebinning algorithm and filtered back projection with the ramp filter cut-off at the Nyquist frequency. The image matrix was 128 × 128 × 159, resulting in a voxel size of 0.776 × 0.776 × 0.796 mm^3^. The 120-min list-mode data were used in the following sequence: eight 15-s, three 60-s, five 5-min, and nine 10-min time frames. The spatial resolution of reconstructed images was 1.63 mm at full width at half maximum [18].

### Data analysis

#### Kinetic analysis

Images were analyzed using the Inveon Research Workplace 4.2. A three- dimensional region of interest (ROI) was manually defined in each mouse for the tumor using the last time frame (110–120 min) of the PET image with a threshold of one-half of the maximum SUV of the tumor. The tumor ROI was automatically copied to every dynamic image to obtain time activity curves (TACs). SUV was calculated from the last time frame (110–120 min) of the PET image using the following equation.

$$ S\mathrm{U}\mathrm{V} = \frac{\mathrm{activity}\ \mathrm{in}\ \mathrm{a}\ 3\mathrm{D}\ \mathrm{tumor}\ \mathrm{R}\mathrm{O}\mathrm{I}\ \left(\mathrm{Bq}/\mathrm{mL}\right)}{\mathrm{injection}\ \mathrm{dose}\ \left(\mathrm{Bq}\right)/\mathrm{body}\ \mathrm{weight}\ \left(\mathrm{g}\right)} $$

**Fig. 1:**
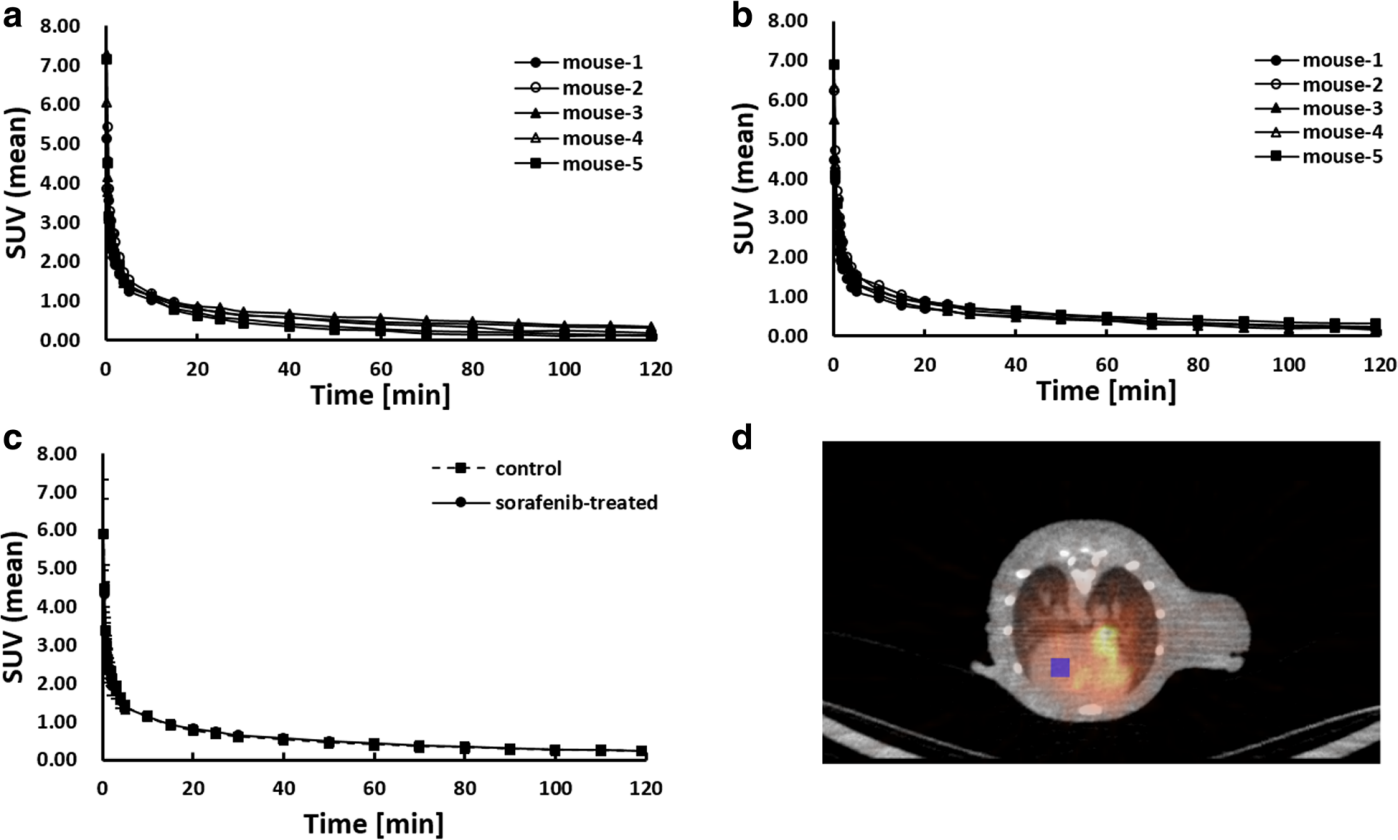
Blood time activity curves (input function) for [^18^F]FLT in the left ventricle. **a** Control group. **b** Sorafenib-treated group. **c** Average of each group (SUV ± SD). **d** Transaxial image of [^18^F]FLT PET across the heart of the mouse at first flame (0–15 s). A cuboid ROI (1.5 × 1.5 × 2.0 mm^3^) was drawn on the left ventricle (LV) region on a CT image and projected to every PET image to obtain the LV TAC (*filled blue region*)

A cuboid ROI (1.5 × 1.5 × 2.0 mm^3^) was drawn on the left ventricle (LV) region on a CT image and projected to every PET image to obtain TACs of [^18^F]FLT in the blood (Fig. 1a–d), which was used as the input function for the modeling analysis [19].

**Fig. 2:**
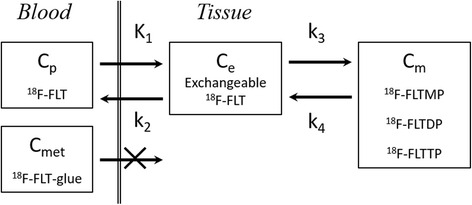
Compartment model of [^18^F]FLT in the tumor tissue. K_1_, k_2_, k_3_, and k_4_ are the kinetic rate constants between the compartments. *Cp* blood concentration of [^18^F]FLT, *Ce* exchangeable [^18^F]FLT concentration in the tissue, *Cm* phosphorylated [^18^F]FLT metabolites in the tissue, *Cmet* concentration of [^18^F]FLT metabolites in the arterial plasma. *FLTMP* FLT-monophosphate, *TLTDP* FLT-diphosphate, *FLTTP* FLT-triphosphate, *FLT-gluc* FLT-glucuronide

A three-compartment model fitting (Fig. 2) was carried out to estimate four rate constants using the TACs of the tumor and the blood [11, 20, 21]. The rate constants of the model represent the forward transport of [^18^F]FLT from blood to tissue (K_1_), the reverse transport (k_2_), the phosphorylation of [^18^F]FLT (k_3_), and the dephosphorylation (k_4_) [20]. The early distribution volume (Vd) of [^18^F]FLT in tissue was also defined as K_1_/k_2_. The kinetic imaging system (KIS), which is an Internet-based kinetic simulation and model fitting program, was used to estimate the values of the rate constants [22]. Correction on the blood volume fraction was included in the modeling of this system. The blood volume was fixed at 0.03 [mL/g]. The amounts of [^18^F]FLT metabolites in the blood were minuscule [23].

### Statistical analysis

Repeated measures analysis of variance (ANOVA) was carried out to assess the differences in TACs between the control and the sorafenib-treated groups. The SUVs of [^18^F]FLT in the tumors and each rate constant were compared using the unpaired *t* test between the control and sorafenib-treated groups. A two-tailed value of *p* < 0.05 was considered significant.

## Results

Figures 1a, b shows the blood clearance curves of the control and sorafenib-treated groups, respectively, which were obtained from the SUVs in the cuboid ROI placed on the LV region (Fig. 1d). There were no significant differences in the input function between the two groups (Fig. 1c).

**Fig. 3:**
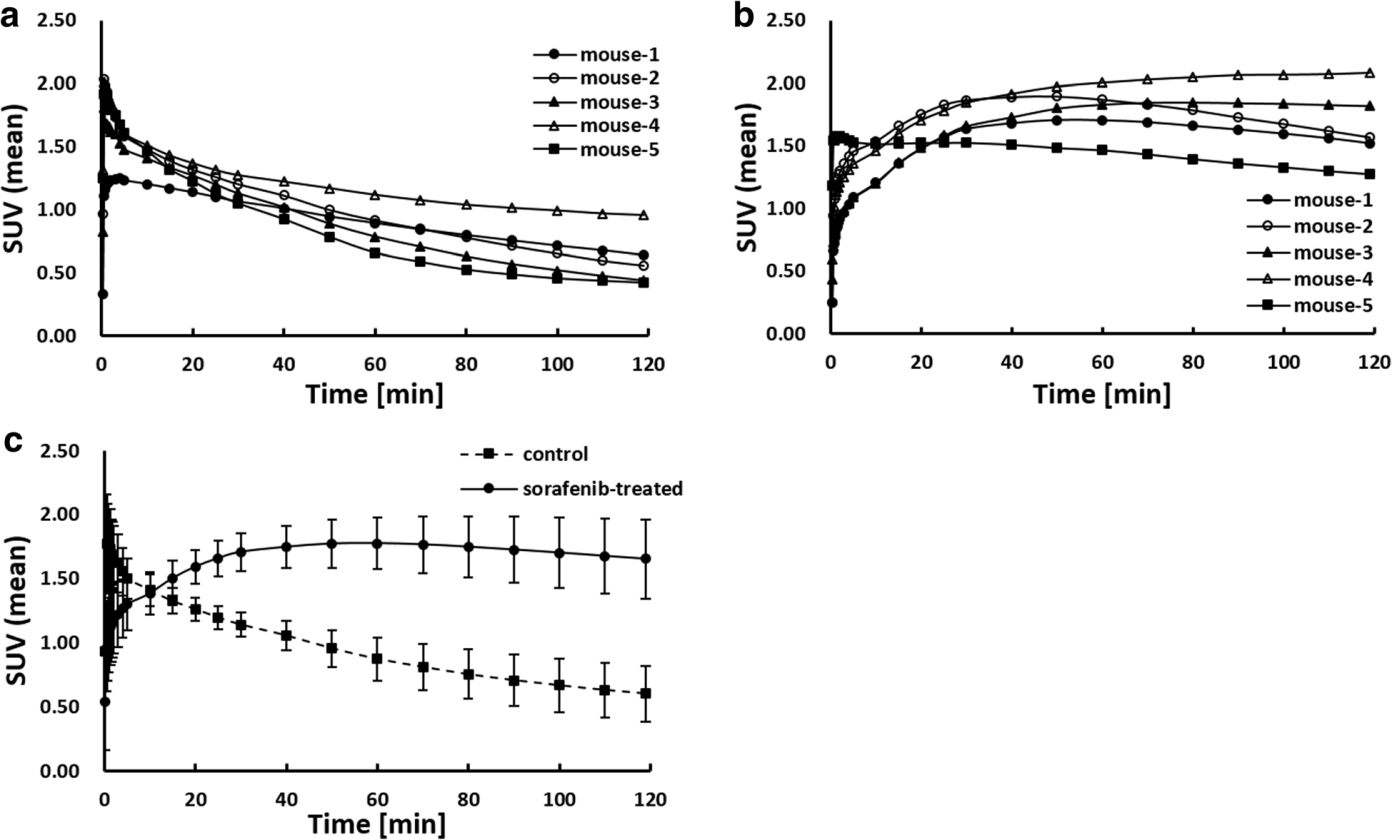
Time activity curves in the tumor following [^18^F]FLT injection. **a** Control group. **b** Sorafenib-treated group. **c** Average of each group (SUV ± SD)

The tumor TACs are shown in Fig. 3. In the control group, the tumor TAC peaked immediately after the injection and then decreased (Fig. 3a, c). In the sorafenib-treated group, the tumor TAC gradually increased with time and reached a plateau (Fig. 3b, c). The dynamic patterns of the tumor [^18^F]FLT level were significantly different between the control and sorafenib-treated groups (*p* < 0.05).

The rate constants (K_1_, k_2_, k_3_, and k_4_) and the Vd (K_1_/k_2_) are summarized in Table 1. K_1_, k_2_, and k_4_ were significantly lower in the sorafenib-treated group than in the control group. k_3_ was significantly higher in the sorafenib-treated group than in the control group. No significant changes were observed in Vd between the control and sorafenib-treated groups.

**Table 1:** Estimated compartment model parameters

	K_1_ (mL/min/g)	k_2_ (1/min)	k_3_ (1/min)	k_4_ (1/min)	Vd (mL/g)
Control	0.547 ± 0.106	1.012 ± 0.284	0.082 ± 0.009	0.037 ± 0.006	0.556 ± 0.073
Sorafenib	0.231 ± 0.050	0.359 ± 0.060	0.111 ± 0.027	0.017 ± 0.003	0.641 ± 0.052
*p* value	<0.01	<0.01	<0.05	<0.01	N.S.

**Fig. 4:**
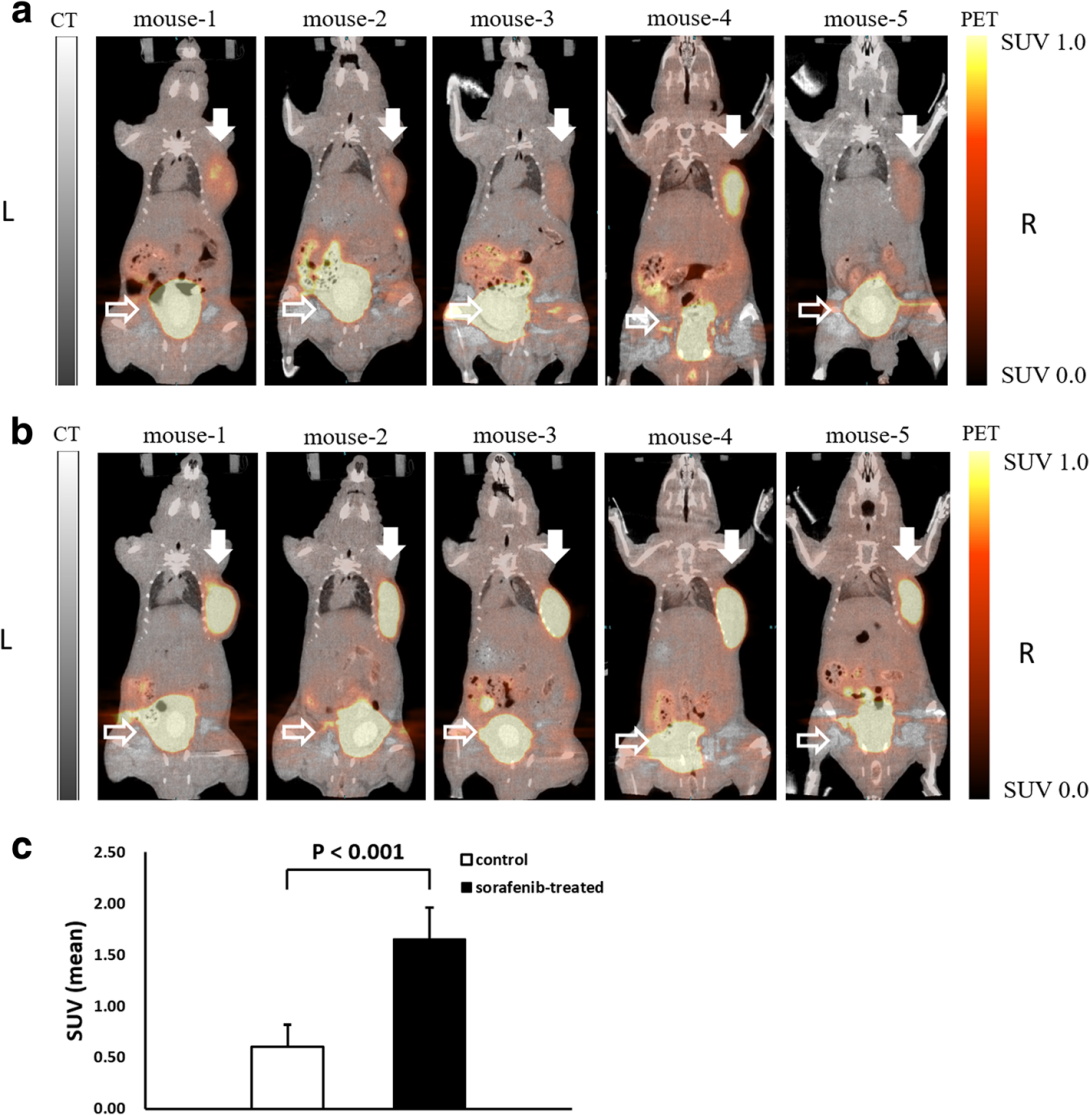
PET images of [^18^F]FLT (*horizontal sections*) at 110–120 min postinjection in the mice bearing the tumor. **a** Control group. **b** Sorafenib-treated group. *Filled arrows* show the tumor region and *blank arrows* show the bladder. Artifacts due to extremely high radioactivity accumulation were observed in the bladder area. **c** SUVs of [^18^F]FLT in the tumor at 110–120 min postinjection

Figure 4a, b shows PET images of [^18^F]FLT (horizontal sections) at 110–120 min postinjection in the mice bearing the tumor. The images clearly show higher radioactivity levels in the tumor regions of the treated mice than those of the control mice. Figure 4c shows the SUVs of [^18^F]FLT in the tumor of the control (0.602 ± 0.216) and sorafenib-treated (1.653 ± 0.309) groups. The SUVs in the tumor of the sorafenib-treated group were significantly higher than those of the control group (Figs. 3 and 4).

## Discussion

In this study, to clarify the reason for the elevated FLT level after sorafenib treatment, which was observed in our previous study [4], we evaluated the dynamic patterns of the [^18^F]FLT level in the tumors by PET. k_3_, which indicates the extent of phosphorylation, was significantly higher in the sorafenib-treated group than in the control group. It is suggested that the increase in the FLT level may be caused by FLT phosphorylation in the tumor after sorafenib treatment.

In this study, we investigated the kinetics of [^18^F]FLT in the RCC tumor and estimated the rate constants (K_1_, k_2_, k_3_, and k_4_). The forward transport (K_1_) and reverse transport (k_2_) were significantly higher in the control group (Table 1). Sorafenib has an anti-angiogenic property; the decrease in vessel density after sorafenib treatment, as shown in our previous histological experiment [24], may reduce the blood flow, resulting in the decrease in K_1_ and k_2_ values. However, no significant changes were observed in the Vd (K_1_/k_2_) between the two groups, suggesting that the change in the blood flow may not affect the accumulation of FLT in the tumor. The higher rate constant k_3_ in the sorafenib-treated group suggests the increased phosphorylation of FLT, which might cause the increased retention of FLT in the tumor. Dephosphorylation may also be responsible for the retention of FLT in the tumor because k_4_ is decreased.

Note that the increase in FLT level was inconsistent with the Ki-67 index, which can be used to evaluate the tumor proliferation in our previous study [4]. Recent studies have revealed the discordance between FLT level and other tumor proliferation markers, for example, Ki-67 index [25, 26]. It is also reported that FLT level does not reflect tumor proliferation but TK1 activity [26–28]. The increase in FLT uptake level is caused by the upregulation of TK-1 activity without an increase in the level of proliferation markers. The present results strongly indicate that the increase in FLT level after sorafenib treatment may be caused by the phosphorylation of FLT. Accordingly, one of the potential causes of the increase in FLT level is the upregulation of TK1 activity that arises from the inhibition of thymidylate synthase (TS). Several studies have shown that FLT uptake level reflects TS inhibition by fluorouracil (5-FU) treatment independent of tumor proliferation changes [29–31]. There are two pathways of thymidine supply for DNA synthesis, the de novo pathway and the salvage pathway. TS and TK1 are critical enzymes in the de novo and salvage pathways, respectively. When the de novo pathway is suppressed, the salvage pathway is upregulated to compensate for this suppression and maintain a certain level of thymidine supply [31, 32]. Thus, TS inhibition or suppression may increase TK1 activity and FLT uptake level [31]. Regarding the effect of sorafenib on the thymidine supply pathways, only one study has suggested the suppression of TS in RCC cells following sorafenib treatment [33]. The increase in FLT uptake level following sorafenib treatment in the present study may have been caused by the TS suppressive effect of sorafenib and the subsequent upregulation of the thymidine salvage pathway.

Our studies also revealed the need for further studies. Analyses of metabolites including phosphorylated FLT in the tumors of control and sorafenib-treated groups may clarify the factors responsible for the increase in FLT level after sorafenib treatment. Evaluation at various time points after sorafenib treatment, particularly long-term observations, will provide additional information on the tumor responses after the treatment.

It should be noted here that there have been several reports on PET studies using [^18^F]FLT and rodents [34, 35]. Honndorf et al. [34] monitored the impact of genistein therapy using the two PET tracers [^18^F]FDG and [^18^F]FLT in vivo in two xenograft mouse models. Rapic et al. [35] investigated the early effects of chemotherapeutic treatment on cancer cell proliferation in a BRAF-mutated colorectal cancer using [^18^F]FLT. These studies focused on the correlation of FLT level and tumor response [3]. In the present study, we evaluated the dynamic patterns of the [^18^F]FLT level in the tumors, to clarify the reason for the elevated FLT level after sorafenib treatment. To date, there have only been limited studies of the therapeutic effect of radiation therapy and chemotherapy on tumor using dynamic PET and compartment model analysis [21, 36, 37]. Moreover, such studies exclusively used FDG in clinical settings. For example, Nishiyama et al. [36] investigated the accumulation of FDG in primary central nervous system (CNS) lymphoma using dynamic PET images and compared with baseline and follow-up kinetic parameters after chemoradiotherapy. They showed that the kinetic analysis, especially with respect to k_3_, might be helpful for monitoring therapeutic assessment. To the best of our knowledge, FLT and kinetic modeling techniques have been utilized to evaluate tumor responses to therapy in a few clinical studies [38–40] and only one experimental study [21]. Pan et al. [21] investigated the change in the rate of cell proliferation in a murine tumor model after radiation therapy using [^18^F]FLT and evaluated the sensitivity of kinetic analysis over semiquantitative measures for monitoring radiation responses. They showed the kinetics of the [^18^F]FLT level and kinetic parameters in the mammary carcinoma cell line, which were altered treatment-dependently 1 day after radiation therapy. In this study, we first applied kinetic analysis and dynamic FLT PET for assessing the tumor responses to molecular-targeted therapy in animal models and succeeded in clarifying the reason for the increase in FLT level after sorafenib treatment. Thus, kinetic analysis and dynamic FLT PET should be useful for assessing the biochemical processes involved in tumor responses to therapy.

There are limitations in this study. ROIs were defined on the LV chamber region to obtain the input function. The LV ROIs were small, and therefore, the input functions may be underestimated due to the partial volume effects (PVEs). The PVEs due to the sizes of the tumor and LV were not corrected in the present study. It may be important to consider the PVEs on our results. The PVEs, however, do not appear to largely affect the comparison of the kinetic parameters between the control and sorafenib-treated mice, as the similar kinetic analysis was performed in both of the groups.

It is important to consider whether similar effects can be obtained even in human studies. Zhang et al. treated the mice bearing human RCC (A498) xenografts with sorafenib [41]. In these experiments, they were able to demonstrate the responses to sorafenib, re-induction of tumor necrosis and associated reduction in tumor perfusion, which were relevant to clinical situation. The results may suggest the relevance of our study in A498 model mice to human studies partly although further studies to clarify whether our results are actually general or not are necessary.

## Conclusions

The dynamic pattern of [^18^F]FLT uptake level in the tumor was significantly changed after sorafenib treatment. The rate constant k_3_, representing FLT phosphorylation, significantly increased after sorafenib treatment, whereas the distribution volume did not change. These findings indicate that the increase in FLT level may be caused by FLT phosphorylation in the tumor after sorafenib treatment in the mice bearing a RCC xenograft. Dynamic PET studies with kinetic modeling could provide improved understanding of the biochemical processes involved in tumor responses to therapy.

## Abbreviations

[^3^H]FLT: [Methyl-^3^H(N)]-3′-fluoro-3′-deoxythythymidine
5-FU: Fluorouracil
AAALAC: The association for assessment and accreditation of laboratory animal care
ANOVA: Analysis of variance
CNS: Central nervous system
FOV: Field of view
KIS: The kinetic imaging system
LV: The left ventricle
PET: Positron emission tomography
PVE: Partial volume effect
RCC: Renal cell carcinoma
ROIs: Regions of interest
SUV: Standardized uptake value
TAC: Time activity curve
TS: Thymidylate synthase
